# Identification of Novel RD1 Antigens and Their Combinations for Diagnosis of Sputum Smear−/Culture+ TB Patients

**DOI:** 10.1155/2016/7486425

**Published:** 2016-01-18

**Authors:** Zhiqiang Liu, Shuang Qie, Lili Li, Bingshui Xiu, Xiqin Yang, Zhenhua Dai, Xuhui Zhang, Cuimi Duan, Haiping Que, Ping Zhao, Heather Johnson, Heqiu Zhang, Xiaoyan Feng

**Affiliations:** ^1^Department of Bio-Diagnosis, Beijing Institute of Basic Medical Sciences, 27 Taiping Road, Haidian District, Beijing 100850, China; ^2^Institute for Medical Devices Control, National Institute for Food and Drug Control, No. 2 Tiantan Xili, Chongwen District, Beijing 100050, China; ^3^Chaoyang District Centre for Disease Control and Prevention, 25 Panjiayuan Huaweili, Beijing 100029, China; ^4^Olympia Diagnostics, Inc., 640 W. California Avenue, Sunnyvale, CA 94086, USA

## Abstract

Rapid and accurate diagnosis of pulmonary tuberculosis (PTB) is an unresolved problem worldwide, especially for sputum smear− (S−) cases. In this study, five antigen genes including Rv3871, Rv3874, Rv3875, Rv3876, and Rv3879 were cloned from* Mycobacterium tuberculosis* (*Mtb*) RD1 and overexpressed to generate antigen fragments. These antigens and their combinations were investigated for PTB serodiagnosis. 298 serum samples were collected from active PTB patients, including 117 sputum smear+ (S+) and sputum culture+ (C+) cases, 101 S−/C+ cases, and 80 S−/C− cases. The serum IgG levels of the five antigens were measured by ELISA. Based on IgG levels, the sensitivity/specificity of Rv3871, Rv3874, Rv3875, Rv3876, and Rv3879 for PTB detection was 81.21%/74.74%, 63.09%/94.78%, 32.21%/87.37%, 62.42%/85.26%, and 83.56%/83.16%, respectively. Furthermore, the optimal result for PTB diagnosis was achieved by combining antigens Rv3871, Rv3876, and Rv3879. In addition, the IgG levels of Rv3871, Rv3876, and Rv3879 were found to be higher in S−/C+ PTB patients than in other PTB populations. More importantly, combination of the three antigens demonstrated superior diagnostic performance for both S−/C+ and S−/C− PTB. In conclusion, the combination of Rv3871, Rv3876, and Rv3879 induced higher IgG response in sputum S−/C+ PTB patients and represents a promising biomarker combination for diagnosing of PTB.

## 1. Introduction

According to the global tuberculosis report released by the World Health Organization in 2013 (http://www.who.int/topics/research/en/), TB is still a worldwide threat to human health. Early and rapid diagnosis is essential for disease control and prevention of spread of the disease, especially for sputum smear− (S−) patients [1, 2].

In the past decades, several methods have been developed for TB diagnosis, including tuberculin skin test (TST), sputum smear and culture test, and nucleic acid amplification test [3–6]. Despite the use of these tests, it is still difficult to diagnose TB quickly and accurately in clinical practice [7]. TST is not a specific test for the diagnosis of active TB, especially in populations that have received* Bacillus* Calmette-Guérin (BCG) or in populations with high rate of exposure to nontuberculous mycobacteria (NTMs) [5]. Acid-fast staining test on sputum smear usually has very low sensitivity (about 30–40%) [8]. As the gold standard for TB diagnosis, sputum smear and culture test is time-consuming that needs 4–8 weeks to obtain result, which is not suitable for rapid TB diagnosis and treatment [9, 10]. PCR-based nucleic acid amplification assays significantly improved rapid diagnosis of TB, but amplification of endogenous inhibition factor of* Mtb* and the lack of reliable quality control have resulted in high false positive and false negative rates [11]. In addition, requirement of dedicated and expensive equipment for PCR assay has hampered its clinical utility, especially in low-income countries. Therefore a rapid test with accurate diagnostic performance is greatly needed for TB diagnosis [11].

A serology blood test, which is easy to perform, low in cost, and easy with testing large amount of samples, represents a promising method for TB diagnosis [12] and has attracted great interests from investigators. In the past few years, encouraging progress has been made in TB serodiagnosis by using specific antigens. A number of* Mtb* antigens have been investigated for their use in TB diagnosis [12–16] and several promising antigens have been identified [17]. However, currently no single antigen is able to achieve sufficiently high sensitivity and specificity simultaneously. The strategy of combining multiple antigens has been shown to significantly enhance diagnostic performance, but none of the combination tests has been reported with satisfactory accuracy for S− TB diagnosis [18, 19]. Consequently, efforts are still desired to identify novel and more effective antigens for TB diagnosis, especially for S− TB cases.

The* Mtb* regions of difference (RD) encode numerous specific antigens and some of them have been extensively studied for TB diagnosis, such as ESAT6 (Rv3875), CFP10 (Rv3874), CFP-21, and MPT-64 [12, 20]. However, they have not been thoroughly investigated as TB-specific antigens. In the present study, our aim was to identify TB-specific antigens and screen combinational antigens with high accuracy for TB diagnosis. We cloned five immunodominant antigens encoded in the* Mtb* RD1 and overexpressed the protein fragments. The IgG levels of the five antigens in different TB populations (S+/C+, S−/+, and S−/C−) were then assessed with indirect ELISA. The accuracy of the tests using the five antigens individually and in combination was evaluated for TB diagnosis.

## 2. Methods

### 2.1. Study Population and Serum Collection

The study participants, including 298 active PTB patients and 94 healthy individuals, were selected consecutively from November 2012 to December 2013 in Beijing Chaoyang District Centre for Disease Control and Prevention. The active PTB was diagnosed based on clinical symptoms, including coughing, fever, coughing up of blood, and pulmonary fibrocavity infiltrates on chest radiograph. For the suspected TB cases, sputum smear and culture test was performed as reported previously [21]. Final TB diagnosis was based on the result of sputum smear and culture test as well as symptomatic improvement after anti-TB therapy. No patient was identified with HIV-1 infection. The patients were further divided into three groups: (1) smear-positive for acid-fast bacilli and culture-positive pulmonary TB (S+/C+ group, *n* = 117), (2) smear-negative and culture-positive pulmonary TB (S−/C+ group, *n* = 80), and (3) smear-negative and culture-negative group (S−/C− group, *n* = 101). Healthy controls were recruited from routine checkup population who showed no clinical symptoms of TB with no prior history of TB infection. In addition, TST and chest radiograph were performed on the healthy controls to exclude potential TB infection.

Before any anti-TB treatment was given to the patients, 3 mL of fasting peripheral venous blood was drawn and collected from each patient. Within 4 hr of blood collection, the samples were centrifuged at 1,200 g for 10 min at 4°C to spin down the blood cells. Then the supernatant was transferred into a new ice cold centrifuge tube and centrifuged as the above. The supernatant was transferred into an RNase/DNase-free tube and stored at −80°C until use.

### 2.2. Cloning, Expression, and Purification of the Recombinant Antigens

The BioSun Version 3.0 software (Developed by the Center of Computational Biology, Beijing Institute of Basic Medical Sciences) was used for B-cell epitope prediction as described in our previous report [17]. The coding sequences of five immunodominant antigens (Rv3871, Rv3874, Rv3875, Rv3876, and Rv3879) were selected and amplified by PCR from* M. tuberculosis* H37Rv genomic DNA with specific end nuclease restriction sites (Xho I and Xba I). The gene fragments were then inserted into the prokaryotic expression plasmid pBVIL1 and overexpressed in* Escherichia coli* HB101 as reported previously [17]. The recombinant proteins were purified by ion exchange and gel filtration, and the protein concentration was determined by the Bradford method (Pierce, Rockford, IL). The purified proteins were aliquoted and stored under −80°C.

### 2.3. Indirect ELISA

Microplates were coated with individual antigens (Rv3871, Rv3874, Rv3875, Rv3876, and Rv3879), respectively, at 5 *μ*g/mL in coating buffer (0.05 M carbonate/bicarbonate, pH 9.6) as described previously [17]. 100 *μ*L diluted serum (1 : 10 in PBST containing 1% BSA) was added to each antigen-coated well. The plates were sealed and incubated at 37°C for 30 min and then washed three times. 100 *μ*L horseradish peroxidase-conjugated anti-human IgG antibody (Sigma, USA) was added to each well followed by 30 min incubation at 37°C in seal. After three times of washing, freshly prepared tetramethylbenzidine (TMB) substrate was added and the plates were incubated for 20 min in room temperature. 0.1 N sulfuric acid was added to the plates and the optical density was measured at 450 nm using an automatic microplate reader (Bio-Rad, USA).

### 2.4. Statistical Analysis

Data processing and graph mapping were performed using GraphPad Prism 4.0 (GraphPad Software, Inc.) and OriginLab software (OriginLab, Inc.). Statistical analysis was performed with SPSS 17.0 software package (SPSS, Inc.). The IgG levels of the different antigens were compared by one-way ANOVA. The diagnostic value of the five antigens with indirect ELISA assay was evaluated by the receiver operating characteristic (ROC) curve analysis. In ROC curve, the optimal operating point (OOP) was determined via Youden's index according to the method reported previously [21]. Briefly, the sensitivity and specificity of each operating point in the ROC curve were automatically calculated using GraphPad Prism 4.0 software, followed by calculation of Youden's index of each point (Youden's index = sensitivity + specificity − 1). By comparing Youden's indices of all points, the maximum values of Youden's index and OOP were determined.

## 3. Results

### 3.1. Generation of Five RD1 Antigens

The B-cell epitopes of five RD1 antigens (Rv3871, Rv3874, Rv3875, Rv3876, and Rv3879) were predicted. Based on the epitope curve, the fragment containing dominant B-cell epitopes with higher peak value was determined. It was 60–96 amino acids (aa) for Rv3871, 26–66 aa for Rv3874, 201–420 aa for Rv3875, 380–510 aa for Rv3876, and 130–220 aa for Rv3879. These fragments were then cloned and expressed, followed by purification of the overexpressed fragments. In the following text, we will call the corresponding fragments as Rv3871, Rv3874, Rv3875, Rv3876, and Rv3879, respectively.

### 3.2. Serodiagnostic Performance of Five RD1 Antigens for Active TB

**Figure 1:**
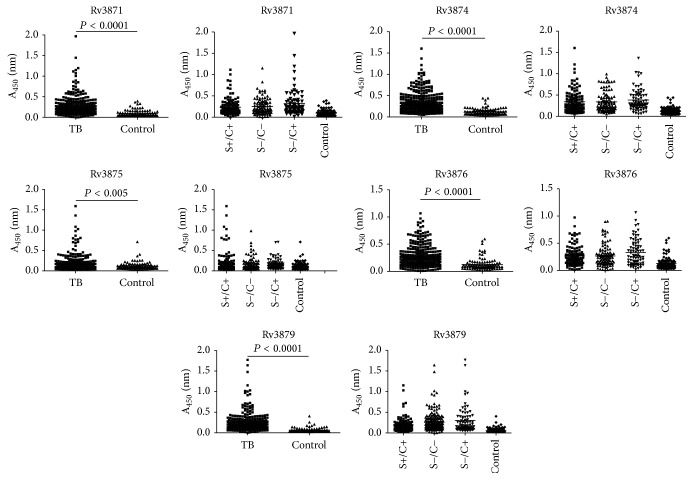
Scattergrams of normalized levels of the five antigens in TB patients and healthy controls. The values in ordinate represent the OD of the measured IgG levels of the antigen in patient serum samples. In each panel, the left scattergram shows IgG distribution in the whole TB population and the healthy control population. The right scattergram shows IgG distribution in three different TB populations (sputum smear+/culture+; sputum smear−/culture+; sputum smear−/culture−) and the control population.

**Figure 2:**
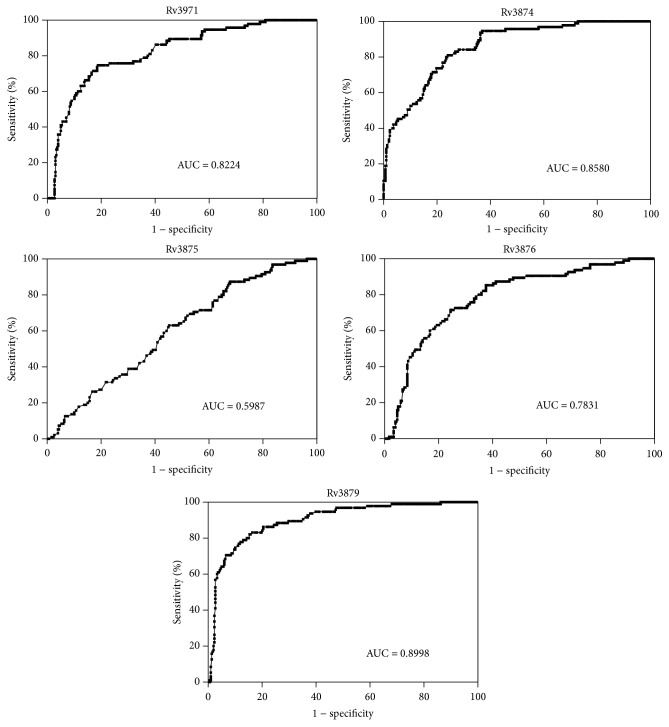
ROC curves analysis of five individual antigens for TB diagnosis.

The serum IgG antibody levels against the five RD1 antigens were measured by ELISA assay in all TB patients consisting of 3 TB groups as well as healthy controls. The results are presented as scattergrams in Figure 1. The serodiagnostic performance of the five antigens for active TB diagnosis was analyzed and ROC curves were mapped, as shown in Figure 2. From ROC analysis, the optimal operating point (OOP) for each antigen at the maximum value of Youden's index was calculated according to a previous report [22]. The cutoff values of the IgG levels against the five antigens were 0.1115, 0.2295, 0.1740, 0.1865, and 0.0915, respectively. At OOP, the overall diagnostic performance of each antigen for active TB was shown in Table 1.

### 3.3. Serodiagnostic Performance of RD1 Antigen Combinations

Based on the diagnostic performance of individual antigens as shown above, various combinations of the five antigens were analyzed in TB diagnosis. We defined the positive detection as detection of IgG of one, two, three, four, and five at least of the five RD1 antigens, respectively. As shown in Table 2, when the diagnostic criteria were set as positive for at least two or three of the five antigens, the results showed 86.91% sensitivity with 81.05% specificity and 70.47% sensitivity with 89.47% specificity, respectively. These two combinations represent optimal diagnostic performance when balancing sensitivity and specificity, which is consistent with Youden's index (the larger Youden's index, the better the diagnostic performance).

In clinical practice, a diagnostic test with ~90% or higher specificity is usually required. Thus, we made an effort to further test various combinations of RD1 antigens for TB diagnosis, aiming to minimize the number of antigens used in the test while improving the diagnostic performance, especially test specificity. First, we analyzed the performance of combining any four RD1 antigens for TB diagnosis. We found that the only possible way to improve the diagnostic performance by combining four antigens over combining five antigens was to define positive as having detected IgG of at least two antigens in the combination. In such case, there were five different combinations. As shown in Table 3, the best diagnostic performance was achieved when Rv3871, Rv3874, Rv3875, and Rv3879 were combined (Youden = 0.7218), with the sensitivity 82.71% and specificity 89.47% (PPV = 96.06%; NPV = 61.15%). Next, we analyzed the performance of combining three antigens. Similarly, we found that the only possible way to improve the diagnostic performance by combining three antigens was to define positive as detection of IgG of at least two antigens. In this case, there were ten different combinations. As shown in Table 4, the best diagnostic performance was achieved when Rv3871, Rv3876, and Rv3879 were combined (Youden = 0.7073), which had sensitivity of 79.53%, specificity of 90.53%, PPV of 96.34%, and NPV of 58.5%.

The above results showed that the optimal combination of three antigens (Rv3871, Rv3876, and Rv3879) had comparable diagnostic performance with the optimal combination of four antigens (Rv3871, Rv3874, Rv3875, and Rv3879 or Rv3871, Rv3875, Rv3876, and Rv3879). However, it is more convenient and cost-effective to use fewer antigens in clinical testing. Thus, the combination of three antigens Rv3871, Rv3876, and Rv3879 is better than the combinations of four antigens for TB diagnosis.

### 3.4. IgG Levels of Antigens Rv3871, Rv3876, and Rv3879 in Smear−/Culture+ TB Patients and the Diagnostic Performance of Their Combination in Different TB Populations

**Figure 3:**
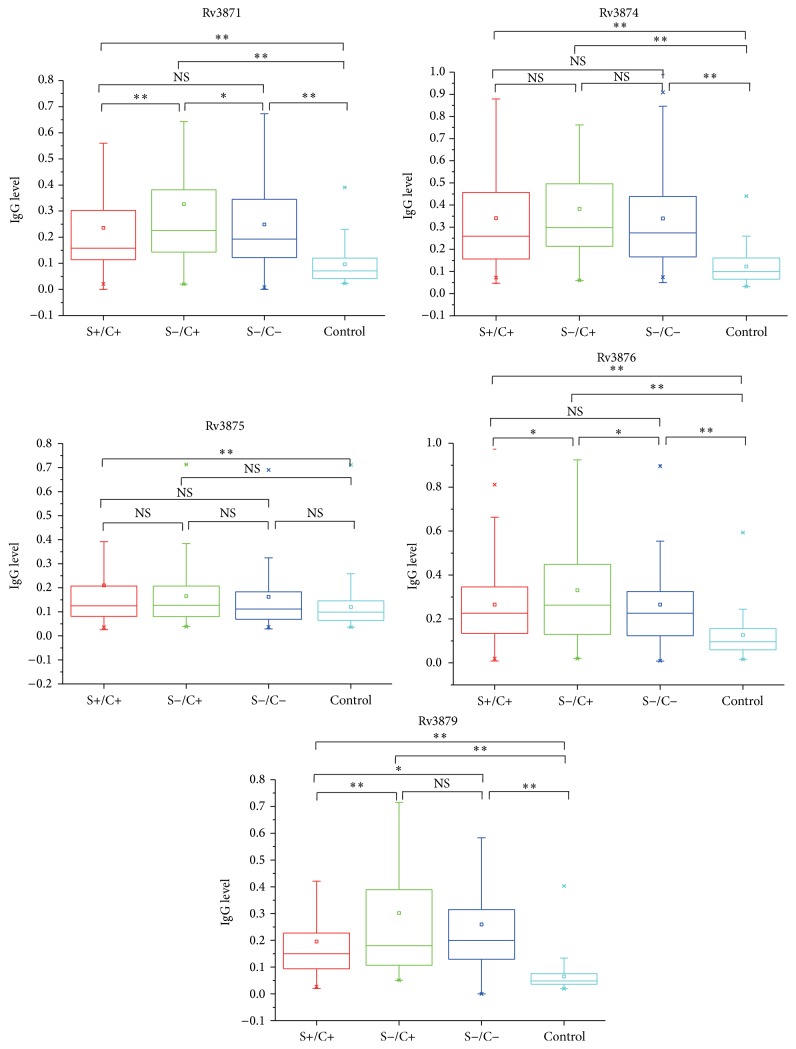
Comparison of IgG levels in healthy control and three TB populations. ^*∗*^ *P* < 0.05, ^*∗∗*^ *P* < 0.01, and NS: no significant difference. The values in ordinate indicate the IgG levels measured as OD value.

We further measured the expression levels of the five antigen-related IgGs in smear+/culture+, smear−/culture+, and smear−/culture− TB populations. As shown in Figure 3, the IgG levels of all five antigens were significantly higher (*P* < 0.01) in three TB populations (S+/C+, S−/C+, and S−/C−) as compared to healthy controls, with the exception of Rv3875, which showed no significant difference between the control and smear− TB population. Interestingly, Rv3871, Rv3876, and Rv3879 IgG levels were especially higher in S−/C+ group than those in the other groups, suggesting that these antigens may have induced higher levels of IgG response in S−/C+ patients than in the other patient groups. Therefore, they may be superior in diagnosis of sputum smear-negative TB patients. Moreover, we analyzed the diagnostic performance of the three-antigen combination in S+/C+, S−/C+, and S−/C− TB populations. As predicted, the three-antigen combination demonstrated high sensitivity and specificity among different TB populations, especially in smear− TB populations (Table 5).

## 4. Discussion

Tuberculosis, which is caused by* Mtb*, is still one of the major health problems around the world. Early TB diagnosis and treatment are important for disease control and prevention [11]. A serological test using antibodies against* Mtb* antigens represents an appealing diagnostic option, which have been investigated previously [1, 23–25]. However, more efforts are still needed to improve the diagnostic accuracy of antigen based serodiagnosis, especially in sputum smear− and culture− TB patients [26, 27]. In this study, we designed and generated five immunodominant antigens encoded in TB RD1 and tested their diagnostic potential individually or in combination in different TB populations. We successfully identified a combination of antigens Rv3871, Rv3876, and Rv3879 and used it with indirect ELISA test to obtain good sensitivity and high specificity in TB diagnosis. More importantly, this antigen combination demonstrated good diagnostic accuracy in both S−/C+ and S−/C− TB patients. To our knowledge, our study is the first thorough investigation of the use of multiple RD1 antigens in TB diagnosis. Our three-antigen combination has achieved the highest accuracy in serodiagnosis of smear− TB cases, which may provide a novel and promising tool for serodiagnosis of TB. In addition, we are the first to report an antigen combination of Rv3871, Rv3876, and Rv3879 with higher expression levels in S−/C+ TB population than in the other TB populations (S+/C+ and S−/C−). Although the underlying mechanism of the differential expression of these antigens in different TB population is still unclear, these antigens may be used for diagnosis of TB cases that cannot be detected by microscopic examination.

It is noteworthy that all of the antigens used in this study were recombinant antigen fragments consisting of only immunodominant epitopes. Using immunodominant fragments instead of the whole protein for antibody detection has been well established in our lab for several years. Previously, we have successfully cloned many antigen fragments of immunodominant epitopes and published several papers about the application of this method to identify biomarkers for disease diagnosis [17, 21, 28]. There are two main advantages of using antigen fragments over using the whole protein. The first advantage is due to the fact that antigen fragments consisting of immunodominant epitopes have superior or comparable binding affinity for antibody, resulting from the removal of the nonbinding redundant sequences, which are present in the whole protein that may block exposure of the immunodominant epitopes. The second advantage is because it is much easier to express smaller protein fragments than large intact proteins. Needless to say, it is necessary to evaluate the immune reactivity of the fragments before using them in a diagnostic application. Indeed, all of these successfully expressed antigen fragments in our study have been evaluated for their reactivity with antibodies. In addition, we have compared several of these antigens with the corresponding whole proteins for their antibody binding activity, including ESAT6 (Rv3875) and CFP10 (Rv3874) that were used in the present study. In serum samples from TB patients, the IgG levels detected by ESAT6 and CFP10 fragments as well as the rate of TB diagnosis were nearly identical to those detected using antigens of the whole proteins. However, the other fragments used in the study were not compared with the whole proteins, since it was difficult to express the whole sequences for some proteins.

At present, TB diagnosis remains a worldwide health problem, especially in developing countries where TB diagnosis is nevertheless mainly dependent on sputum smear and sputum culture due to their low cost [1, 29, 30]. However, low sensitivity of sputum smear usually results in a lot of undetected TB cases, while time-consuming sputum culture cannot give rapid diagnosis [31]. Consequently, both of the methods are inadequate for clinical practice. Serological test based on* Mtb*-specific antigens is relatively simple and low cost, suitable as a diagnostic and screening test, especially in developing countries. The key to developing a serological test is to identify sensitive antigen markers (single or multiple), which has encouraged many investigators to explore the field [32–34]. In our study, we evaluated five novel immunodominant antigens and found that most of them could be used in serodiagnostic test with a comparable sensitivity to sputum culture except for one antigen Rv3875, demonstrating their significant potential in clinical applications. It is noteworthy that all of these antigens are encoded in TB RD1. The region is usually deleted in avirulent strains and therefore is specific for pathogenic strains and can avoid interference by BCG inoculation. This may explain the superiority in specificity of these antigens. The superior specificity of antigens in TB RD1 has been confirmed by a previous report [35].

In development of a diagnostic test, it is necessary to determine an optimal serum dilution for IgG detection and define diagnostic criteria. In our preliminary experiment, we performed serial dilution tests to determine the optimal serum dilution. We found that 1 : 10 dilution was optimal in our experimental system. However, 1 : 10 dilution may not be optimal in other systems, because the experimental conditions may vary among different research groups. In addition, individual variance of background signal may exist with the serum dilution due to the nonspecific protein binding, which should be considered before using our test method. In our study, we investigated whether the nonspecific protein binding could be excluded by thorough washing after serum incubation. We found that the background OD can be consistently reduced to minimum after three times of washing, and this can be achieved in different serum samples. In other words, no significant individual variance of background OD was observed after three times of washing. Therefore, in our current study, all of the plates were washed at least three times.

Among the five antigens investigated in the present study, although several of them as a single antigen could obtain comparable performance to sputum culture, none of them had sufficiently high sensitivity and specificity suitable for diagnosis in clinic. This may be due to the high heterogeneity of humoral response upon* Mtb* infection [36]. In fact, many single antigens have been tested in TB diagnosis before and up till now, no single antigen has been reported to have consistently satisfactory sensitivity and specificity. Due to the heterogeneity of* Mtb* infection, it is generally considered that a single antigen may never achieve satisfactory diagnostic performance. Therefore, serodiagnosis based on multiple antigens was recommended by many studies in recent years. Deng et al. combined three* Mtb*-related antigens in diagnosis, with the diagnostic criteria defined as positive detection of any one of the three antibodies. The test was shown to have about 90% sensitivity and ~80% specificity [18]. In another study by Kalra et al., four antigens encoded in RD1 and RD2 were combined and tested in TB diagnosis, resulting in an enhanced sensitivity up to ~80% [12]. These studies proved the superiority of multiantigen based diagnosis. Despite these studies, an accurate serodiagnostic test for smear− TB has not been reported. An issue with the combined antigen strategy is the balance of specificity and sensitivity, which were usually inversely related. In multiple-antigen based diagnosis, the sensitivity normally would decrease with the number of positive markers used in detection criteria, while the specificity would increase. In the present study, we explored all possible combinations of the five antigens (from single antigens to combining two, three, four, and five antigens) and analyzed the diagnostic performance with different criteria. At the end, antigens Rv3871, Rv3876, and Rv3879 were demonstrated to be an optimal combination, achieving 79.53% sensitivity and 90.53% specificity (PPV = 96.34%; NPV = 58.5%). More importantly, combination of these three antigens showed similarly accurate diagnostic performance in smear- and culture-negative TB populations. The high accuracy in smear- and culture-negative TB populations is of great clinical significance. This is especially important in low-income countries, where it can be used as a low cost point of care test for TB diagnosis, or it can be used as a test complementary to existing microscopic examination. Interestingly, our study further revealed that the IgG levels of Rv3871, Rv3876, and Rv3879 were higher in S−/C+ TB cases than in S+/C+ and S−/C− cases, suggesting that higher IgG responses against these antigens have been induced. The result may explain why this antigen combination has demonstrated good performance for smear− TB cases. This phenomenon suggests a feasible strategy to test biomarkers and diagnostic methods to be used for diagnosis of TB undetected by sputum smear or culture tests. However, our present study has not discovered the underlying mechanism of this phenomenon, which deserves further in-depth investigation.

In TB diagnosis, sputum culture was considered the gold standard test. However, in clinical practice, actually there are some TB cases which are sputum culture-negative, which means some TB cases could not be detected by sputum culture. Nevertheless, these sputum culture-negative patients have TB-related symptoms, and more importantly, they can experience symptomatic improvement after receiving antituberculous therapy. Therefore, these patients should be diagnosed as having TB. Some clinicians and investigators even think that symptomatic improvement after antituberculous therapy should be a reliable and complementary diagnostic criterion to the standard sputum culture test. In this study, evaluation of the diagnostic performance of combinational biomarkers for sputum culture-negative TB cases was one of our study aims; therefore we included some sputum culture-negative TB cases, which were diagnosed based on clinical symptoms and symptomatic improvement after antituberculous therapy. In addition, all of the participants were confirmed as HIV (human immunodeficiency virus) negative. Actually, in HIV prevalent countries, a lot of patients have coinfection of TB and HIV. In these TB/HIV coinfected cases, rapid TB diagnosis is even more difficult due to the increased smear-negative patients. The World Health Organization (WHO) has recommend an algorithm for the diagnosis of smear-negative TB in HIV prevalent areas, which, however, was far from satisfactory with a low positive predictive value of 0.34 (95%, CI 0.26–0.43) [37]. In fact, it is still a challenging task to measure antibody levels in sputum smear-negative, culture-positive TB patients associated with other bacterial burdens [38]. Our antigen combination test may provide a promising method for TB diagnosis in HIV prevalent areas and therefore deserves further investigation in TB/HIV coinfected patients.

In summary, our study has thoroughly investigated five novel RD1 antigens in TB diagnosis and identified Rv3871, Rv3876, and Rv3879 as combination antigens for serodiagnosis of TB. We have also demonstrated that their IgG levels were especially high in S−/C+ TB patients. Importantly, the three combination antigens may have achieved the most accurate diagnostic performance in all S+/C+, S−/C+, and S−/C− TB populations as compared with previously reported tests in the field. In addition, the identification of high IgG levels of these antigens in S−/C+ TB patients suggests a feasible testing strategy by using these antigens for detecting TB patients who otherwise cannot be diagnosed by microscopic sputum smear or culture tests.

## Figures and Tables

**Table 1:** Diagnostic performance of individual antigens.

Antigens		Rv3871		Rv3874		Rv3875		Rv3876		Rv3879	
	*N*	+	−	+	−	+	−	+	−	+	−

TB	298	242	56	188	110	96	102	186	112	249	49
Control	95	24	71	5	90	12	83	14	81	16	79

Sensitivity		81.21%		63.09%		32.21%		62.42%		83.56%	
Specificity		74.74%		94.74%		87.37%		85.26%		83.16%	
Youden		**0.5595**		**0.5783**		**0.1958**		**0.4768**		**0.6672**	
PPV		90.98%		97.41%		88.89%		93.0%		93.96%	
NPV		55.91%		45%		44.86%		41.97%		61.72%	

PPV: positive predictive value, NPV: negative predictive value, and Youden: Youden index = sensitivity + specificity – 1.

**Table 2:** Diagnostic performance of five antigen combinations. The positive detection was defined as positive with different number of antigens as shown.

Positive antigens		One^*∗*^		Two^*∗*^		Three^*∗*^		Four^*∗*^		Five^*∗*^	
	*N*	+	−	+	−	+	−	+	−	+	−

TB	298	288	10	259	39	210	88	146	152	56	242
Control	95	41	54	18	77	10	85	1	94	0	95

Sensitivity		96.64%		86.91%		70.47%		48.99%		18.79%	
Specificity		56.84%		81.05%		89.47%		98.95%		100%	
Youden		**0.5348**		**0.6796**		**0.5994**		**0.4794**		**0.1879**	
PPV		87.54%		93.50%		95.45%		99.32%		100%	
NPV		84.38%		66.38%		49.13%		38.21%		28.19%	

*∗* indicates the least number of antigens defined for positive detection.

**Table 3:** Diagnostic performance of combinations of 4 antigens. The positive detection was defined as positive with at least two antigens.

Combined antigens		Rv71-74-75-76^*∗*^		Rv71-74-75-79		Rv71-74-76-79		Rv71-75-76-79		Rv74-75-76-79	
	*N*	+	−	+	−	+	−	+	−	+	−

TB	298	224	74	244	54	255	43	243	55	228	70
Control	95	10	85	10	85	16	79	11	84	11	84

Sensitivity		75.17%		82.71%		86.44%		82.37%		77.29%	
Specificity		89.47%		89.47%		83.06%%		88.42%		88.42%	
Youden		**0.6464**		**0.7218**		**0.6950**		**0.7079**		**0.6571**	
PPV		95.73%		96.06%		94.1%		95.67%		95.4%	
NPV		53.46%		61.15%		64.75%		60.43%		54.55%	

^*∗*^Rv71-74-75-76 represents the combination of Rv3871, Rv3874, Rv3875, and Rv3876 for TB diagnosis. The other combinations are also abbreviated including Rv71-74-75-79, Rv71-74-76-79, Rv71-74-76-79, Rv71-75-76-79, and Rv74-75-76-79.

**Table 4:** Diagnostic performance of combinations of 3 antigens. The positive detection was defined as positive with at least two antigens.

Combined antigens	*N*	TB	Control	Sensitivity	Specificity	Youden	PPV	NPV
		298	95					
Rv71-76-79	+	237	9	79.53%	90.53%	**0.7006**	96.34%	58.5%
	−	61	86					

Rv71-75-79	+	222	10	74.5%	89.47%	**0.6397**	95.69%	52.8%
	−	76	85					

Rv71-75-76	+	187	10	62.75%	89.47%	**0.5222**	94.92%	43.37%
	−	111	85					

Rv71-74-79	+	239	11	80.20%	88.42%	**0.6862**	95.6%	58.74%
	−	59	84					

Rv71-74-76	+	216	12	72.48	87.37%	**0.5985**	94.74%	50.3%
	−	82	83					

Rv71-74-75	+	189	6	63.42%	93.68%	**0.5710**	96.92%	44.95%
	−	109	89					

Rv75-76-79	+	192	9	64.43%	90.53%	**0.5496**	95.52%	44.79%
	−	106	86					

Rv74-76-79	+	224	13	75.93%	86.32%	**0.6225**	94.51%	52.56%
	−	74	82					

Rv74-75-79	+	186	3	62.42%	96.84%	**0.5926**	98.41%	45.1%
	−	112	92					

Rv74-75-76	+	164	4	55.03%	95.79%	**0.5082**	97.62%	40.44%
	−	134	91					

**Table 5:** Diagnostic performance of the combination of Rv3871, Rv3876, and Rv3879 in different TB populations. The positive detection was defined as positive with at least two antigens.

	*N*	Pos.	Neg.	Sensitivity	Specificity	Youden	PPV	NPV
S+/C+	117	89	28	76.07%	90.53%	**0.6660**	90.81%	75.43%
S−/C+	80	68	12	85%^*∗*^	90.53%	**0.7553**	88.3%	87.76%
S−/C−	101	80	21	79.21%^*∗*^	90.53%	**0.6974**	89.89%	80.37%
Control	95	9	86					

^*∗*^S+/−: sputum smear +/− TB, C+/−: sputum culture +/− TB, Pos.: positive, Neg.: negative.
